# A Comparative Analysis of Grip Strength Evaluation Methods in a Large Cohort of Aged Mice

**DOI:** 10.1002/jcsm.70050

**Published:** 2025-08-27

**Authors:** Giorgia Bigossi, Serena Marcozzi, Maria Elisa Giuliani, Giovanni Lai, Beatrice Bartozzi, Fiorenza Orlando, Laura Gerosa, Amir Mohammad Malvandi, Diana Putavet, Esmée Bouma, Peter L. J. de Keizer, Giovanni Lombardi, Marco Malavolta

**Affiliations:** ^1^ Advanced Technology Center for Aging Research and Geriatric Mouse Clinic IRCCS INRCA Ancona Italy; ^2^ Advanced Technology Center for Aging Research IRCCS INRCA Ancona Italy; ^3^ Experimental Animal Models for Aging Unit, Scientific Technological Area IRCCS INRCA Falconara Marittima (AN) Italy; ^4^ Laboratory of Experimental Biochemistry & Advanced Diagnostics IRCCS Ospedale Galeazzi‐Sant'Ambrogio Milan Italy; ^5^ Cleara Biotech B.V Utrecht the Netherlands; ^6^ Center for Molecular Medicine, Division of Laboratories, Pharmacy and Biomedical Genetics University Medical Center Utrecht Utrecht the Netherlands; ^7^ Department of Clinical and Molecular Sciences (DISCLIMO) Università Politecnica Delle Marche Ancona Italy

**Keywords:** biogerontology, geriatric mouse model, grip strength, musculoskeletal aging, preclinical research

## Abstract

**Background:**

Grip strength is a key functional marker of musculoskeletal aging, widely used to assess sarcopenia. In preclinical research, multiple measurement methods are often combined to enhance reliability, but standardization remains challenging. To improve measurement robustness, we previously developed a composite strength score (SS5) that integrates five different grip strength tests into a single variable. While SS5 provides a comprehensive evaluation, its implementation is time‐consuming, limiting feasibility in large‐scale studies. In this study, we also examine two simplified composite scores, SS2 and SS3, as potential streamlined alternatives. Additionally, although normalizing grip strength to body weight is widely used, its appropriateness in geriatric mouse models has never been formally validated.

**Methods:**

Forelimb grip strength was assessed in a cohort of 160‐aged C57BL/6J mice using five methods: Weight Lift Tests (Deacon protocol with sponge weights and a modified version with metal wire weights), the Cage Lift Test and the Grip Strength Meter (trapeze bar and grid). Additionally, a cross‐sectional group of 173 mice was analysed to assess the correlation between grip strength and muscle size. Each method was evaluated for its correlation with age, ability to detect sex differences, variability and association with muscle size.

**Results:**

All methods strongly correlated with age (−0.518 ≤ r_s_ ≤ −0.306). The Grip Strength Meter (trapeze bar) and modified Deacon method were the most effective in detecting sex differences (*p* < 0.001). While all methods correlated with muscle size (0.153 ≤ r_s_ ≤ 0.332), the modified Deacon method and Grip Strength Meter showed the strongest associations. The mean coefficient of variation (CV%) ranged from 7% to 17%, demonstrating good repeatability. Notably, despite being widely used, normalization of grip strength to body weight was found to introduce bias in geriatric mice, as age‐related weight loss distorts strength assessments. Absolute values proved to be a more reliable measure. To improve efficiency while maintaining reliability, we developed two new composite scores (SS2 and SS3) by integrating a subset of methods from SS5. These scores preserved the strong correlation with age observed in SS5 while reducing the number of required tests, enhancing feasibility.

**Conclusions:**

Combining multiple grip strength assessments improves measurement reliability in aging studies. The newly proposed SS2 and SS3 scores provide a streamlined yet robust alternative to SS5, improving standardization and facilitating future comparisons in preclinical sarcopenia research. Our findings also challenge the routine normalization of grip strength to body weight in geriatric mice, emphasizing the importance of using absolute values to avoid bias.

## 1 Introduction

Musculoskeletal aging is a critical and complex process, characterized by a gradual decline in muscle mass, strength and functional capacity [1, 2]. One of its most prominent manifestations is sarcopenia, an age‐related loss of muscle mass and function that has a considerable impact on the overall health and well‐being of the elderly population [3, 4]. Hand grip strength (HGS) is now recognized as an essential clinical marker for evaluating muscle function [5, 6, 7]. HGS has been strongly associated with key health outcomes, including mobility, multimorbidity and mortality risk [8, 9, 10]. Its simplicity and noninvasiveness make it an ideal measure for both clinical and preclinical research settings [11].

Translating this measure into preclinical studies provides valuable insights into the assessment of muscular health and enables precise monitoring of physical frailty phenotype progression, facilitating treatment responses in aging models [12, 13, 14]. Among preclinical models, the geriatric mouse model is crucial for aging studies and its age‐related conditions, like sarcopenia, since it closely reflects the physiological and pathological changes observed in elderly humans [15, 16]. Additionally, the relatively short lifespan of the most widely used strains of mice, C57BL6 and HET3 (median lifespan around 29–30 months) [17], allows the execution of longitudinal studies, which are of utmost importance when studying processes underlined by a temporal decline such as sarcopenia [18]. Despite its importance, current methods for measuring grip strength in the mouse model vary widely, and their relative effectiveness in detecting age‐related muscle decline remains inadequately explored. To improve the robustness of grip strength assessment, we previously developed a composite strength score (SS5) that integrates five different grip strength tests into a single variable [19]. This approach was designed to enhance measurement reliability by reducing variability associated with individual methods. The assessment encompasses the Weight Lift Tests (with one version utilizing sponge weights according to the original Deacon protocol [20, 21] and a modified version employing weights made of metal wires [19]), the Cage Lift Test [22] and the gold standard method, the Grip Strength Meter, using both trapeze bar and grid tools [23]. Notably, several studies [24, 25] have reported grip strength normalized by body weight. However, despite its widespread use, no studies have formally investigated whether this approach introduces bias in geriatric mouse models, where age‐related weight loss may distort strength assessments.

This study evaluates five grip strength assessment methods in mice, assessing their effectiveness in detecting age‐related muscle decline in longitudinal studies. The methods are compared based on four criteria: correlation with age, sex discrimination, intragroup and interbatch variability and association with muscle size. These criteria were used to refine measurement techniques, improving reproducibility and applicability in preclinical models of musculoskeletal aging. Furthermore, to explore streamlined alternatives, this study also investigated two composite scores SS2 and SS3 derived from subsets of the individual methods, assessing their performance relative to the composite score SS5.

## 2 Materials and Methods

### 2.1 Experimental Animals

All experiments were conducted in accordance with the European Community Council Directive 2010/63/EU. The protocols were approved under current Italian legislation (D. Lgs. n. 26/2014) by the Organismo Preposto al Benessere Animale (OPBA, Animal Care and Health Committee) of IRCCS INRCA and the General Directorate of Animal Health and Veterinary Drugs of the Italian Ministry of Health. The study was authorized under no. 392/2019‐PR (aimed at study frailty in a longitudinal cohort of mice). The mice were divided into two batches based on the enrolment period: *n* = 109 (2021–2022, Batch 1) and *n* = 51 (2022–2023, Batch 2). An additional cross‐sectional group (*n* = 173, enrolled between 2022 and 2023) was included to specifically evaluate the correlation between grip strength measurements and muscle size.

The mice were housed at the INRCA Geriatric Mouse Clinic under controlled conditions in a Specific Pathogen‐Free (SPF) environment.

### 2.2 Sample Size Estimation

Although the present study was conducted using a dataset originally generated for broader investigations on aging and frailty, we performed an a priori sample size estimation using G*Power (v. 3.1.9.7) to verify its adequacy for the primary outcomes related to grip strength.

To estimate the expected effect size, we reanalysed our previously published dataset of 546 aged C57BL/6J mice [19], in which the Strength Score trajectories were modelled using a generalized linear model including age, sex, their interaction and cohort as fixed effects.

The effect of sex was significant (*F*(1, 599) = 10.610, *p* = 0.001), corresponding to a partial *η* ^2^ = 0.0174 (effect size *f* = 0.133). A power analysis based on this value using a repeated measures ANOVA design (6 timepoints, α = 0.05, power = 0.80) indicated a required minimum sample size of approximately 100 animals.

Similarly, the effect of age was highly significant (*F*(1, 599) = 32.364, *p* < 0.001), with a partial *η* ^2^ = 0.0518 (effect size *f* = 0.227), leading to an estimated required sample size of approximately 127 animals for adequate power.

As the present study included 160 animals (76 males and 84 females), it exceeds both thresholds and thus provides sufficient statistical power to detect sex‐ and age‐related effects across all grip strength measures and derived scores evaluated in the analysis.

### 2.3 Measurement of Grip Strength and Muscle Size

During a monthly screening, measurements were systematically documented using various forelimb grip strength assessment methods, following a training phase consisting of two consecutive monthly follow‐up sessions. All were conducted during the afternoon hours of the light cycle (11 AM to 5 PM).

Mice included in the study were monitored monthly throughout their lifespan, except for the subgroup designated for organ harvesting.

The five grip strength measurements used in this study (see Figure S1 for descriptive pictures) are as follows:

1 Weight lift test

- Deacon: Based on the weights lift method described initially by Deacon in 2013 and revised by Malavolta et al. [20, 21, 22]; we used a set of weights consisting of balls made of tangled fine gauge stainless steel wire, typically used to prevent scale buildup in domestic kettles, as previously reported. The weights for grip assessment included 20, 33, 46, 52.5, 59, 62.5, 72 and 85 g [21].
- Modified Deacon: The weight lift method kept similar to the Deacon method but used wire weights with the same diameter as the bars of the mouse cage (2 mm) [19]. The weights of this set of wire weights were exactly the same as reported above.

For the two weight lift tests proposed, the grip strength was measured using the method of Deacon et al., with adaptations from Malavolta et al. (2019) to better detect strength alterations in geriatric mice. Briefly, mice were held by the middle/base of the tail and lowered to grasp a starting weight (20 g) with their forepaws. A successful hold was defined as maintaining the weight for 3 s. If the mouse failed, the time held was recorded. If successful, the mouse was tested on progressively heavier weights until it failed. Malavolta et al.'s adaptation introduced intermediate weights (52.5 and 62.5 g) around the critical 59‐g threshold to enhance discrimination of strength changes in older mice. The final score was calculated as reported by Deacon et al. [20].

2 Cage lift test

The mouse is gently held by the tail and lifted to the top of an empty cage placed on a scale with a fast response. The measurement is based on the negative weight recorded on the scale. The average of the two most negative peaks from around 10 attempts is recorded and expressed in grams (g) [19].

3 Grip strength meter

Forelimb grip strength was assessed using a digital force gauge (Ugo Basile, Varese, Italy) with two grasping tools: (a) grid strength meter equipped with a plastic grid and (b) bar strength meter using a trapeze bar. Mice were gently held by the tail and lowered until their forepaws firmly grasped a grid and bar connected to the digital force gauge. Once the grip was secured, the mice were positioned horizontally to the apparatus, and a smooth, steady pull was applied until the forepaws released the bar or grid [26].

When the mouse grasped the grid or the bar, the maximum pull force, measured in grams (g), was recorded directly by the digital force transducer, part of an apparatus with a digital display and a grasping tool [27, 28].

When the mouse underwent 5 or 6 measurements expressed in grams (g), the two highest and most consistent readings were selected for subsequent analysis.

In the cross‐sectional group of animals, involving 173 mice (100 males and 73 females) with an age range at enrolment of 26 to 30 months (mean age: 26.21 ± 2.09 months; males: 26.69 ± 2.11 months; females: 25.55 ± 1.88 months), each animal underwent a single measurement session, during which muscle size (MS) was assessed using digital callipers, a noninvasive in vivo method that effectively evaluates skeletal muscle dimensions, expressed in centimetres (cm). This group was included to assess the correlation between MS and the five grip strength measurements.

### 2.4 Computation of Grip Strength Variables and CV%

#### 2.4.1 Grip Strength Score

Grip strength measurements described above were transformed into grip strength scores ranging from 0 to 1, where 0 corresponds to the minimum recorded measurement and 1 corresponds to the sex‐specific maximum grip strength observed in our sample and computed as the value of the 95th percentile. We used the 95th percentile to measure maximum grip to avoid bias due to potential outliers. This transformation allows for a more intuitive understanding of the data, as a value of 0.5, for example, would indicate a 50% grip strength compared to the maximum recorded in the population. It should be noted that some individual data points may exceed 1, as these represent grip strengths that are above the 95th percentile. This method of expressing the data provides a clear and concise way to understand the distribution of grip strength within our sample. To improve the robustness of our analysis, we introduced fixed minimum thresholds for each method, calculated across the entire population. This method of expressing data ensures consistency and comparability in grip strength assessments across different methods. The five computed variables will be defined as Deacon Grip Strength Score (DGs), Modified Grip Strength Score (MGs), Cage Lift Strength Score (CGs), Grid Strength Score (GGs) and Bar Strength Score (BGs).

#### 2.4.2 Grip Strength Normalized Body Weight

Body weight‐adjusted strength was calculated by dividing the measured strength value in grams by the body weight of the mice [24, 25]. The resulting values were then transformed into GripBW (body weight) scores, ranging from 0 to 1, with the computed variables transforming to Deacon Grip normalized to Body Weight (DGbw), Modified Grip normalized to Body Weight (MGbw), Cage Lift normalized to Body Weight (CGbw), Grid Strength normalized to Body Weight (GGbw) and Bar Strength normalized to Body Weight (BGbw).

#### 2.4.3 Strength Scores

The five grip strength scores were used to compute a comprehensive score, the Strength Score 5‐Methods (SS5) as already reported by Marcozzi et al. [19]. Briefly, the SS5 was calculated as the arithmetic mean of the individual scores obtained from the DGs, MGs, CGs, GGs and BGs ranging from 0 (*Minimal strength*) to 1 (*Optimal strength*). Additionally, we developed two other new comprehensive strength scores: the strength score 3‐methods (SS3), calculated as the arithmetic mean of the scores obtained from MGs, CGs and BGs, and the strength score 2‐methods (SS2), calculated as the arithmetic mean of the scores obtained from MGs and BGs. SS3 and SS2 were preliminarily selected as the best combinations of 2 or 3 parameters based on the criteria described.

#### 2.4.4 CV% for Grip Measurements

The intragroup variability was calculated as the coefficient of variation (CV%), determined by the ratio of the standard deviation to the mean (SD/mean). This calculation used 5 values obtained from monthly repeated measures in a subset of animals aged 24 to 26 months. Interbatch variability was evaluated by calculating the CV% for each grip strength measurement and comparing it between experimental batches (Batch 1 and Batch 2), using five values derived from monthly repeated measures in a subset of animals aged 24 to 26 months. The selected age range provided the highest number of data points for calculating the coefficient of variation (CV%). Although measurements extended up to 35 months, this range was also chosen because it offered more consistent data, enabling a reliable assessment of variability with reduced influence from the increased variability and reduced sample size observed in later stages of aging.

To compare which method exhibited the least variation, we calculated the mean CV% as the mean of the CV% calculated at 24–26 months for each of the five strength measurement methods.

### 2.5 Statistical Analysis

Differential survival patterns were estimated by Kaplan–Meier using the Log‐Rank test and Cox regression, considering possible confounder variables (sex and experimental batch).

The distribution of the variables was assessed using the Kolmogorov–Smirnov and Shapiro–Wilk tests.

For longitudinal data analysis, a generalized linear mixed model analysis (GLMM) was used to account for repeated measures on the same subjects and the progressive reduction in sample size over time [29].

This approach was employed to analyse the age‐related decline in strength for each method, taking into account the study's longitudinal design. The identifier of each mouse, sex, type of batch and age were included in the model. The linear models were developed assuming a linear distribution with the identity link function. The Satterthwaite approximation with a robust estimator was used for unbalanced data and violation of the assumptions.

This approach accounts for the within‐subject correlations inherent in repeated measurements, thereby improving the accuracy and reliability of effect estimates. The identifiers of each mouse—sex, age and experimental batch were—included in the model as fixed effects, serving as covariates to directly estimate their influence on the grip strength measurements. Additionally, the interaction between sex and age was modelled to assess whether age‐related changes in strength differ between males and females. Temporal correlation was modelled using an ARMA covariance structure. Overall, this modelling framework offers a robust and flexible method to analyse complex longitudinal data with repeated measures and multiple covariates included as fixed effects.

The presence of tumours and abdominal distension was systematically documented throughout the study to account for their possible impact on grip strength and included as a confounding factor in the GLMM analysis of individual grip strength methods and composite scores SS5, SS3 and SS2.

Spearman's correlation and linear mixed model (LMM) were used to compare the age‐related decline in muscle strength among the five methods. The LMM was specifically designed to account for repeated measures, with grip strength scores as the dependent variable and age in months as the covariate. Spearman's correlation analysis was performed to assess the relationship between MS and age, analysing the entire cohort and stratifying the data by sex. All statistical analyses were performed with SPSS (v.26).

The statistical analysis of the mean of coefficients of variation (mean CV%) among the five methods in the entire cohort and across different batches was conducted using GraphPad Prism software (version 7.0, San Diego, CA, USA). Intragroup variability was assessed using a one‐way ANOVA followed by Tukey's post hoc test for multiple comparisons, while interbatch variability was evaluated using a two‐way ANOVA followed by Tukey's post hoc test for multiple comparisons to determine *p*‐values.

## 3 Results

### 3.1 Population Study Overview

A cohort of 160 C57BL/6J mice (Table S1), equally balanced by sex and age at enrolment (mean age: 24.64 ± 0.48 months; males: 24.67 ± 0.47 months; females: 24.62 ± 0.48 months), was used in this study. They were divided into two distinct batches: Batch 1 (*n* = 109) included mice enrolled in 2021–2022; Batch 2 (*n* = 51) consisted of mice enrolled in 2022–2023.

Monthly follow‐up assessments were conducted from enrolment throughout the mice's lifespan until natural death or humane suppression due to severe illness, excluding 13 mice used for organ explants (as control in other studies) after 4–5 measurements. The average number of follow‐up assessments was 6.84 ± 2.99 (6.80 ± 3.00; females: 6.87 ± 2.98). The mean age at death was 29.16 ± 3.54 months (males: 28.99 ± 3.33; females: 29.31 ± 3.77). Figure S2 shows the survival curve of the study population. The number of recorded data at each age is provided in Table S2.

### 3.2 First Criteria: Age Correlation

**FIGURE 1:**
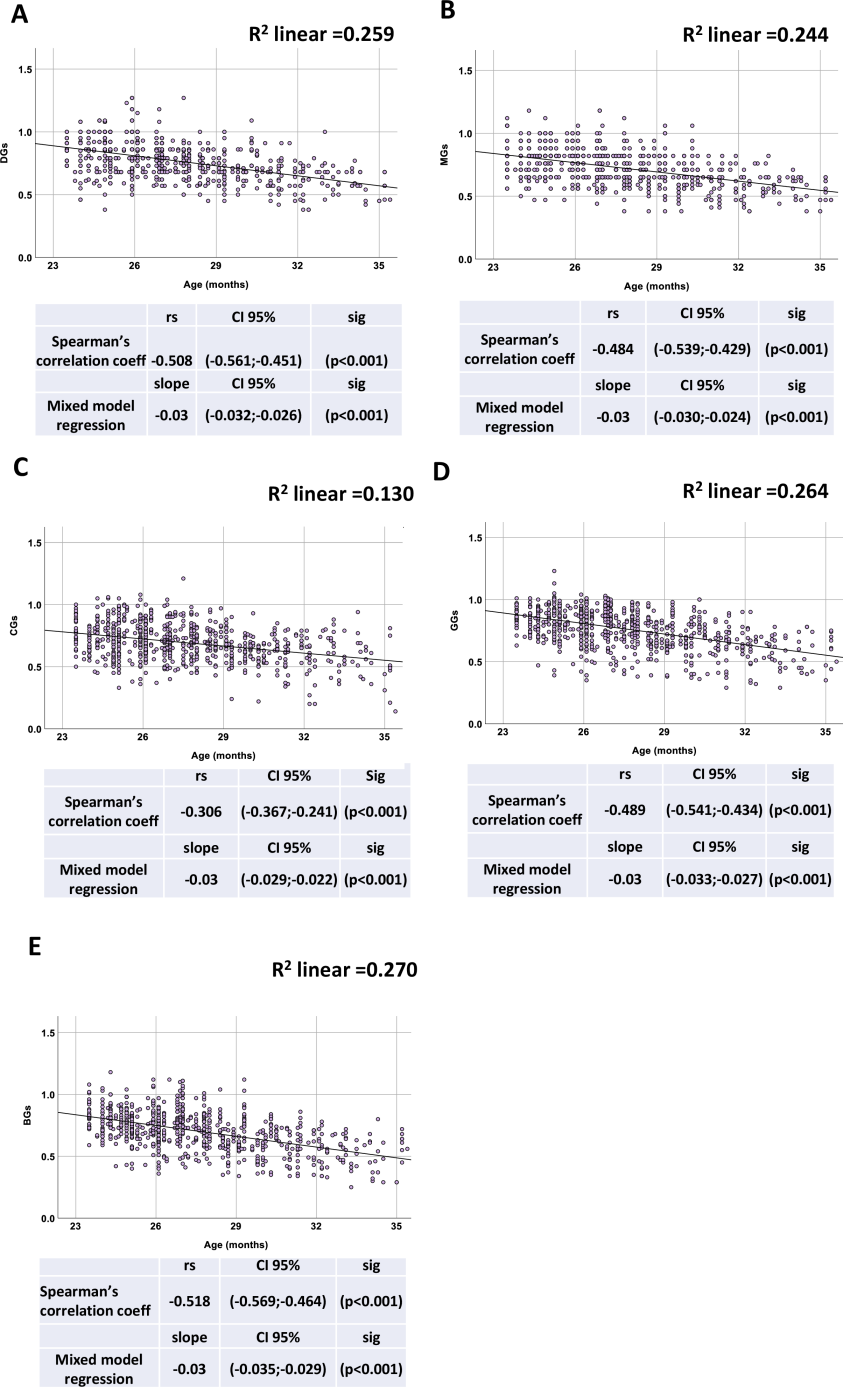
Correlation with age of the five grip strength assessment methods. Correlation was evaluated in a cohort of C57BL/6J mice (*n* = 160), monitored longitudinally for a total of 782 grip strength measurements. Panels A–E show scatter plots relating age to scores obtained with the Deacon Grip Strength (DGs), Modified Grip Strength (MGs), Cage Lift Strength (CGs), Grid Strength meter (GGs) and Bar Strength meter (BGs) methods, respectively. Each plot displays the full dataset with corresponding linear regression lines and *R* ^2^ values. Statistical analysis was conducted using two approaches: (1) Spearman's rank correlation coefficient (r_s_) with 95% confidence intervals (CIs) and *p*‐values; and (2) linear mixed‐effects models (LMMs), which account for repeated measurements in a subset of animals, providing slope estimates with 95% CIs and *p*‐values. All methods demonstrated a significant inverse correlation with age.

The longitudinal design of our study allowed us to track forelimb strength in the same cohort of mice throughout their lifespan, using five different assessment methods. Data were transformed into scores ranging from 1 (*Highest strength*) to 0 (*Lowest strength*) to compare the performances obtained with the five grip strength methods. The results shown in Figure 1 demonstrated a statistically significant and consistent inverse Spearman's correlation between all grip strength scores and age, indicating their ability to accurately detect subtle age‐related physical decline in mice, with no significant differences among the parameters. These findings were further validated through a LMM analysis for repeated measures, which confirmed a progressive decline in all strength scores with age, again with no significant differences among the parameters (Figure 1). The raw grip strength measurements in association with age, along with the regression line and *R* ^2^ coefficient, are presented in Figure S3. Hence, all strength scores are equally associated with age, with no significant differences observed (see Table 1).

**TABLE 1:** Comparison of different strength measurement methods based on the first evaluation criterion: correlation with age. The table summarizes the correlation between grip strength measurements and age using two statistical approaches. The top section reports Spearman's rank correlation coefficients (r_s_) with corresponding 95% confidence intervals (CI). The bottom section reports slopes and 95% CIs derived from linear mixed‐effects model (LMM) regression, which accounts for repeated measurements in the longitudinal dataset. Confidence intervals for both analyses were estimated to assess the precision of the associations. Grip strength was measured using the following methods: Deacon Grip Strength Score (DGs), Modified Grip Strength Score (MGs), Cage Lift Strength Score (CGs), Grid Strength Score (GGs) and Bar Strength Score (BGs).

Statistical method	Grip strength measurements	Coefficient (r_s_)	CI 95%
Spearman's rank correlation	DGs	−0.508	(−0.561, −0.451)
	MGs	−0.484	(−0.539, −0.429)
	CGs	−0.306	(−0.367, −0.241)
	GGs	−0.489	(−0.541, −0.434)
	BGs	−0.518	(−0.569, −0.464)
Statistical method	Grip strength measurements	Slope	CI 95%
Mixed model regression	DGs	−0.03	(−0.032; −0.026)
	MGs	−0.03	(−0.030; −0.024)
	CGs	−0.03	(−0.029; −0.022)
	GGs	−0.03	(−0.033; −0.027)
	BGs	−0.03	(−0.035; −0.029)

### 3.3 Second Criteria: Sex Discrimination in Strength Measurements

We conducted an in‐depth analysis of each grip method using generalized mixed models for repeated measures to investigate further how the five strength measurement methods reveal differences between males and females over time.

Based on preclinical literature, which highlights differences between male and female mouse models, similar to observations in humans [30, 31, 32], i.e., a greater grip strength for males than for females, we aimed to assess whether these methods accurately reflect such sex‐specific differences. The analysis demonstrated a consistent trend across males and females, both exhibiting a significant linear decline in all grip strength measurements as age increased.

**FIGURE 2:**
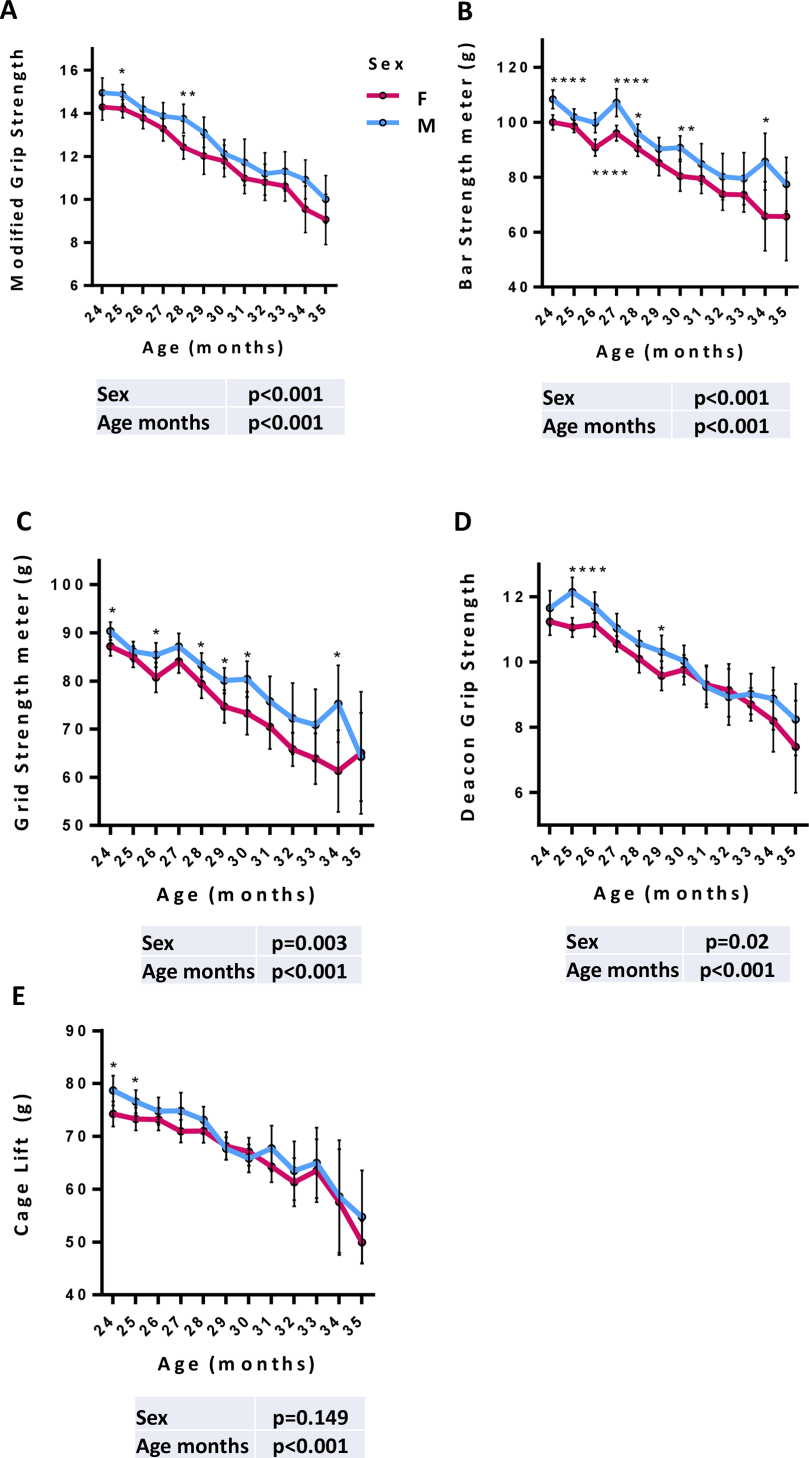
Sex discrimination of the five grip strength assessment methods. Quantitative estimation of the five strength measurements in a cohort of C57BL/6J mice (*n* = 160) monitored longitudinally for a total of 782 measurements. Panels A–E show the trajectories of Modified Grip Strength (MG), Bar Strength meter (BG), Grid Strength meter (GG), Deacon Grip Strength (DG) and Cage Lift (CG), respectively, plotted as a function of age (in months) for male (blue line) and female (red line) mice. Values are presented as model‐derived mean estimates with 95% confidence intervals, obtained from a generalized linear mixed model (GLMM) analysis for longitudinal data. The model included sex, age (in months) and experimental batch as fixed effects. *P*‐values for the fixed effects of sex and age are reported in the insets. Asterisks indicate statistically significant differences by pairwise comparison between sexes at specific timepoints (*p* < 0.05, *p* < 0.01, *p* < 0.001 and *p* < 0.0001). Among the tested methods, MG and BG demonstrated the highest sensitivity in detecting sex‐related differences in muscle strength during aging.

Among the methods, MG and BG emerged as the most robust parameters for detecting sex differences in grip measurements (*p* < 0.001), followed by GG (*p* = 0.003) and DG (*p* = 0.02). In contrast, the CG was not affected by sex (*p* = 0.149), as indicated by the comparable results observed between male and female subjects (Figure 2 and Table S3).

### 3.4 Third Criteria: Grip Strength Variability

**FIGURE 3:**
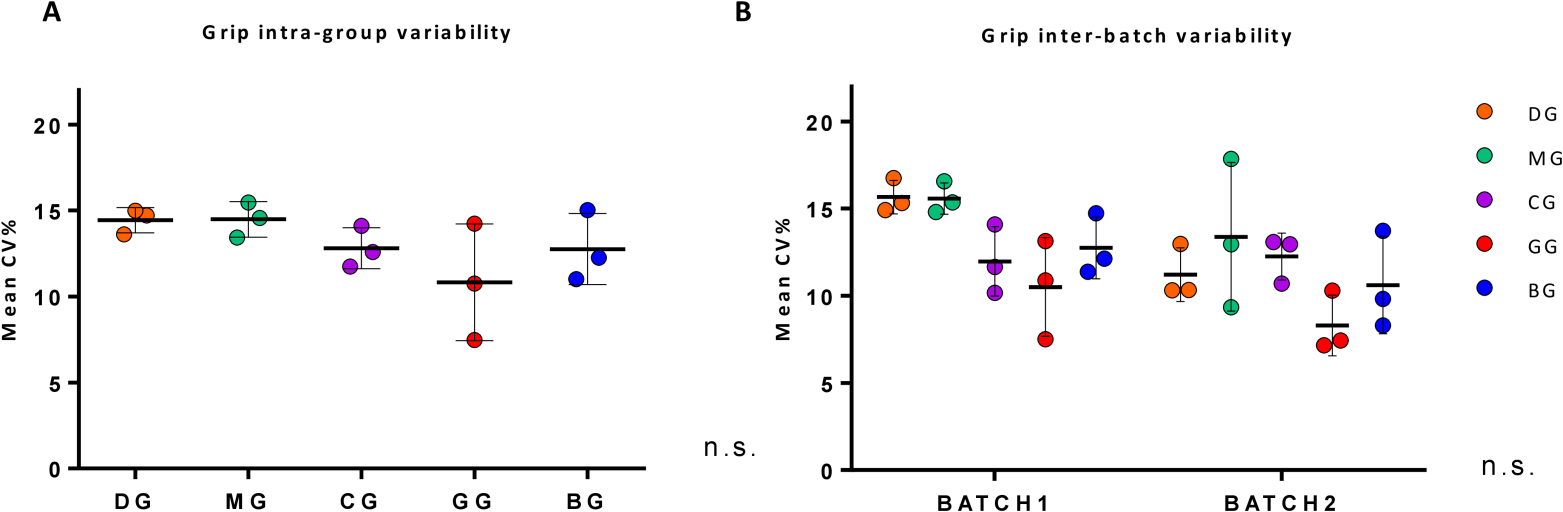
Intragroup and interbatch variability for the five grip strength measurements. (A) Intragroup variability was evaluated by calculating the coefficient of variation (CV%) for each grip strength method at three distinct timepoints: 24, 25 and 26 months of age. The analysis was conducted in a longitudinal cohort of 160 C57BL/6J mice, with 90 mice assessed at 24 months, 150 at 25 months and 126 at 26 months, for a total of 366 measurements. Each dot represents the CV% for a given method at a specific timepoint, and black horizontal bars indicate the mean CV% across the three ages. (B) Interbatch variability was assessed by calculating CV% values separately for two independent experimental batches. Each point represents the CV% of a given method at one of the three timepoints within each batch. Horizontal black bars represent mean CV% for each method within each batch. Statistical analysis was performed using one‐way ANOVA for both intragroup (Panel A) and interbatch (Panel B) comparisons. No significant differences were found among methods (Panel A) or between batches (Panel B) (n.s., not significant). Grip strength was measured using the following five methods: Deacon Grip Strength (DG, orange), Modified Deacon (MG, green), Cage Lift (CG, purple), Grid Strength (GG, red) and Bar Strength (BG, blue). Among all methods, CG and GG showed lower intragroup variability, while BG exhibited good consistency across both intra‐ and interbatch comparisons.

To evaluate the variability of the data, the intragroup coefficient of variation (CV%) was calculated for each of the five grip strength measurements in a subpopulation of animals aged 24 to 26 months. The results revealed the CV% reported in Table 2, with consistent strength measurements across all age classes at 24, 25 and 26 months. To compare which method exhibited the least variation, we calculated the mean CV% in the 24–26 months range for the five strength measurement methods, treating the CV% as a continuous variable. No significant differences between the methods were observed (Figure 3A). To assess interbatch variability, the mean CV% for the five strength measurement methods was compared for the two experimental batches (Table S4). The results showed no significant differences (Figure 3B), indicating no batch‐specific effects on the repeatability of the strength measurements.

**TABLE 2:** Intragroup variability for each grip method. The table reports the mean, standard deviation (SD) and coefficient of variation (CV%) for each grip strength method, along with the number of mice (*n*) assessed at 24, 25 and 26 months of age, in a longitudinal cohort of 160 C57BL/6J mice. A total of 366 measurements were collected across the three timepoints: 90 at 24 months, 150 at 25 months and 126 at 26 months. Grip strength was measured using the following methods: Deacon Grip Strength (DG), Modified Grip Strength (MG), Cage Lift (CG), Grid Strength meter (GG) and Bar Strength meter (BG).

Age months	Variable	DG	MG	CG	GG	BG	(*n*)
24	(Mean ± SD)	11.21 ± 1.65	14.59 ± 2.26	76.30 ± 8.96	88.97 ± 6.65	104.47 ± 11.49	
	CV%	14.71	15.47	11.74	7.47	11.00	90
25	(Mean ± SD)	11.51 ± 1.73	14.59 ± 1.73	74.15 ± 10.46	86.42 ± 9.30	102.07 ± 12.50	
	CV%	14.99	13.42	14.10	10.76	12.25	150
26	(Mean ± SD)	11.38 ± 1.54	14.07 ± 2.05	73.45 ± 9.24	83.44 ± 11.88	96.19 ± 14.44	
	CV%	13.61	14.56	12.58	14.24	15.02	126

### 3.5 Fourth Criteria: Strength‐Muscle Size Correlation

In the cross‐sectional group of animals, involving 173 mice (100 males and 73 females) with an age range at enrolment of 26 to 30 months (mean age: 26.21 ± 2.09 months; males: 26.69 ± 2.11 months; females: 25.55 ± 1.88 months), we conducted a correlation analysis using Spearman coefficients to examine the association between Grip Strength score and MS.

**FIGURE 4:**
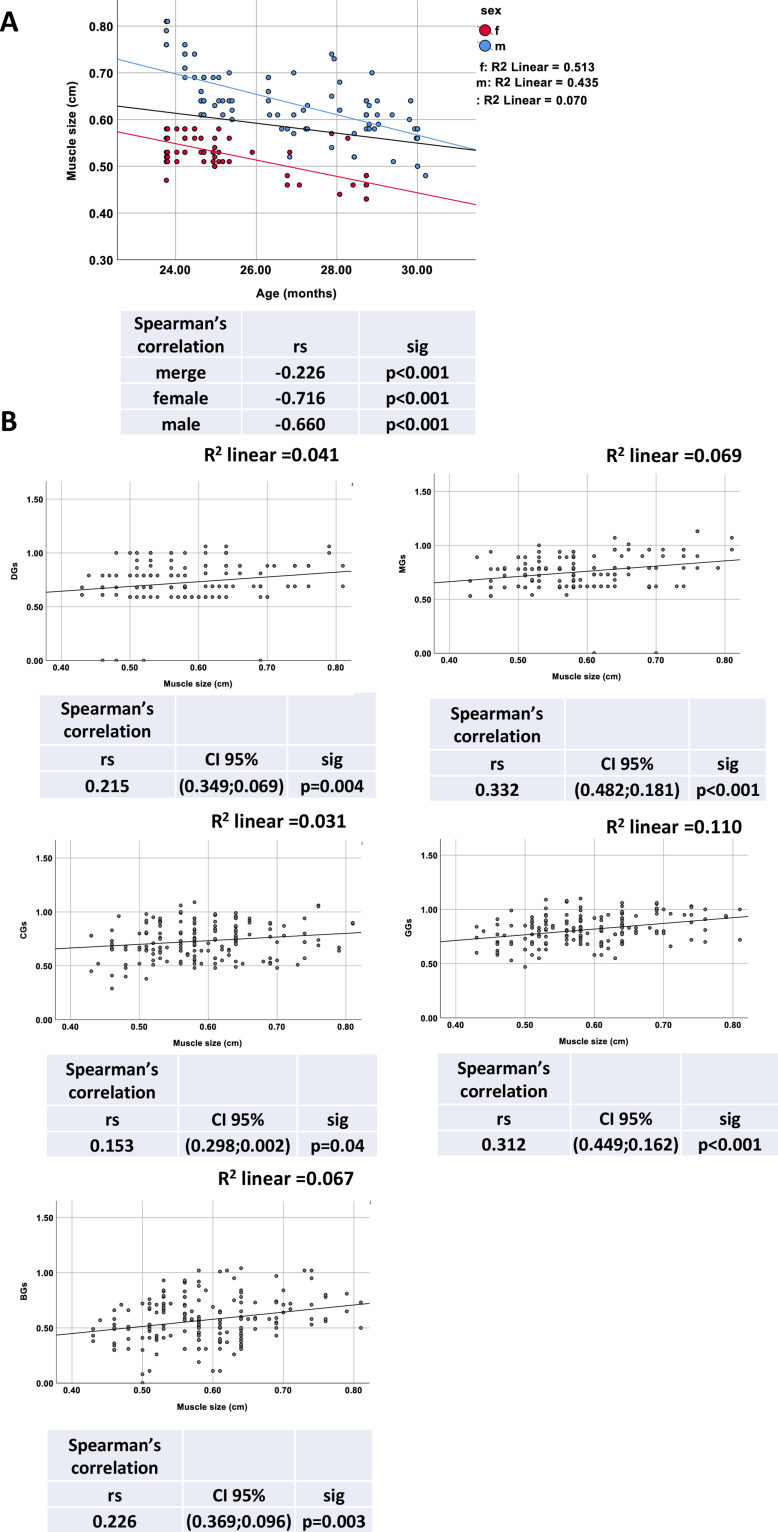
Correlation of muscle size and the five grip strength measurements. Muscle size (MS) was measured in a cross‐sectional cohort of C57BL/6J mice, including 100 males and 73 females, each assessed at a single timepoint. (A) Correlation between muscle size and age, shown for the entire population (black line), and separately for male (blue) and female (red) mice. Linear regression (*R* ^2^) and Spearman's correlation coefficients (r_s_) with significance levels (*p*‐values) are reported for each subgroup. (B) Correlation between muscle size and grip strength assessed using five methods: Deacon Grip Strength Score (DGs), Modified Grip Strength Score (MGs), Cage Lift Strength Score (CGs), Grid Strength Score (GGs) and Bar Strength Score (BGs). Each scatter plot includes linear regression (*R* ^2^) and results of Spearman's correlation analysis, reported with 95% confidence intervals (CIs) and *p*‐values.

Analysis revealed that MS shows a gradual linear decline with age, but this reduction is less pronounced than grip strength measurements. Spearman correlation analysis confirmed an inverse association between MS and age in months, with a coefficient of −0.181 (*p* < 0.017) for the entire cohort, −0.701 (*p* < 0.001) for males and −0.580 (p < 0.001) for females (Figure 4A).

Importantly, our results showed a statistically significant direct correlation between all GS and MS (Figure 4B). Specifically, the CGs exhibited the weakest correlation with MS (rs = 0.156, *p* = 0.04) followed by DGs score (rs = 0.215, *p* < 0.004) and BGs score (rs = 0.226, *p* < 0.003). GGs and MGs scores exhibited the strongest correlations with MS (rs = 0.312, *p* < 0.001 and rs = 0.332, *p* < 0.001, respectively), indicating their high reliability in detecting subtle physical decline as muscle mass and strength deteriorate with age.

### 3.6 Comparison Between GripBW and Grip Strength Score

Studies conducted on younger or adult animals up to a maximum age of 26 months have utilized strength measurements as a normalized value for body weight (strength/BW) [24, 25] To investigate whether this approach applies to geriatric cohorts, we also computed all the strength scores adjusted by body weight and compared the Spearman's rank correlation coefficient and linear regression models for measures repeated concerning age (Table 3). The results indicate that GripBW exhibits a lower Spearman's rank correlation coefficient across all grip strength scores, suggesting that the correlation between GripBW and age is significantly weaker (Figure S4). The results of the mixed model regression for repeated measures showed significant differences between GripBW and Grip Strength scores, except for the MG, which appears to be minimally influenced by the weight variable.

**TABLE 3:** Comparison between absolute grip scores and grip scores normalized to body weight. The table summarizes the comparison between absolute grip strength values and those normalized to body weight, using two statistical approaches. The top section reports Spearman's rank correlation coefficients (r_s_) with corresponding 95% confidence intervals (CI). The bottom section reports slopes and 95% CIs derived from linear mixed‐effects model (LMM) regression, which accounts for repeated measurements in the longitudinal dataset. Confidence intervals for both analyses were estimated to assess the precision of the associations. A significant difference between absolute and normalized scores is indicated . Grip strength was measured using the following methods: Deacon Grip Strength (DG), Modified Grip Strength (MG), Cage Lift (CG), Grid Strength meter (GG) and Bar Strength meter (BG).

Statistical method		Absolute grip scores coefficient (rs) IC 95%		GripBW coefficient (r_s_) CI 95%	
Spearman's correlation	DG	−0.508	(−0.561, −0.451)	−0.350	(−0.418; −0.283)*
	MG	−0.484	(−0.539, −0.429)	−0.346	(−0.401; −0.282)*
	CG	−0.306	(−0.367, −0.241)	−0.157	(−0.223; −0.091)*
	GG	−0.489	(−0.541–0.434)	−0.296	(−0.362; −0.234)*
	BG	−0.518	(−0.569, −0.464)	−0.350	(−0.409; −0.289)*

Statistical method		Absolute grip score slope IC 95%		GripBW slope CI 95%	
Mixed model regression	DG	−0.03	(−0.032; −0.026)	−0.02	(−0.025; −0.019)*
	MG	−0.03	(−0.030; −0.024)	−0.02	(−0.026; −0.019) n.s.
	CG	−0.03	(−0.029; −0.022)	−0.02	(−0.022; −0.015)*
	GG	−0.03	(−0.033; −0.027)	−0.02	(−0.023; −0.016)*
	BG	−0.03	(−0.035; −0.029)	−0.02	(−0.027; −0.020)*

Figure S5 illustrates the age‐related decline in weight, grip strength score and GripBW using a representative aged mouse exhibiting pronounced weight loss. The trends differ markedly, with GripBW not showing an evident age‐related decline. This suggests that normalizing grip strength by body weight is not a suitable approach for accurately assessing grip strength in geriatric studies, especially when aging significantly induces alteration in body weight.

### 3.7 Comparative Analysis of Strength Score

To evaluate the performance of different strength scoring methods, we compared the SS5 published by Marcozzi et al. [19] with two alternative models: SS3 and SS2. The SS3 method integrates the CG, an easily replicable test suitable for any laboratory setting, with the MG, which provides a progressive strength assessment that effectively differentiates between sexes and correlates more closely with MS, and the BG, which exhibits the highest correlation coefficient with age and effectively distinguishes between sexes. In contrast, the SS2 focuses solely on the MG and BG tests, allowing for a more pronounced evaluation of sex diversity in strength.

A generalized mixed model analysis for repeated measures was applied to assess their correlation with age and ability to discriminate between sexes over time.

**FIGURE 5:**
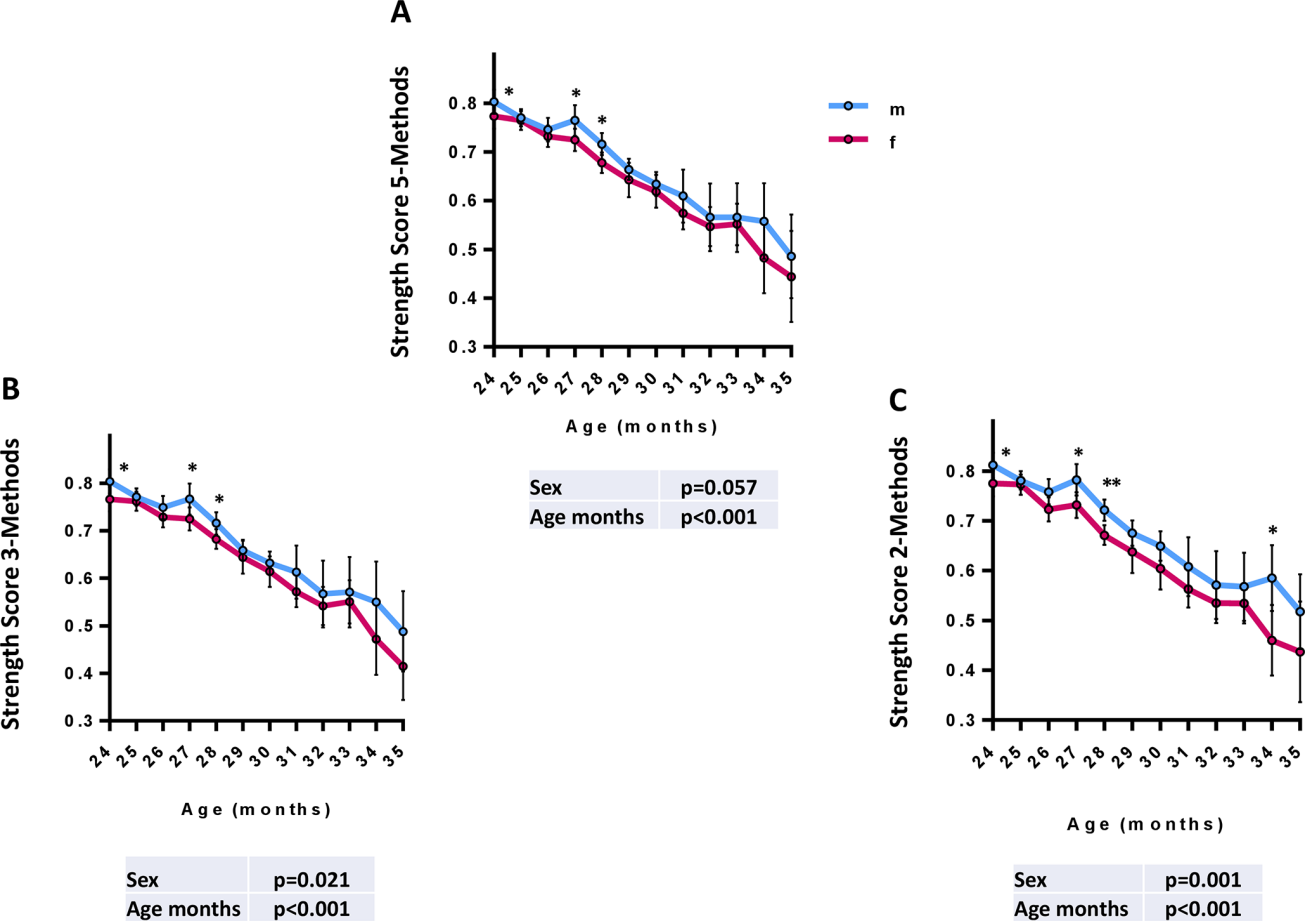
Association of age with strength score 5‐methods (SS5), strength score 3‐methods (SS3) or strength score 2‐methods (SS2). C57BL/6J mice (*n* = 160) were monitored longitudinally, with repeated grip strength measurements collected over time, resulting in a total of 782 observations. Composite strength scores were calculated based on different combinations of grip strength tests: (A) SS5, a five‐method score including Deacon Grip Strength (DG), Modified Grip Strength (MG), Cage Lift (CG), Grid Strength meter (GG) and Bar Strength meter (BG) (as previously defined by Marcozzi et al.); (B) SS3, incorporating three methods (MG, CG and BG); (C) SS2, based on two methods (MG and BG). The scores are plotted as a function of age in male (blue) and female (red) mice. Data are shown as model‐derived mean estimates with 95% confidence intervals, obtained using Generalized Linear Mixed Model (GLMM) analysis for longitudinal data. Sex, age (in months) and batch were included as fixed effects. *P*‐values for sex and age are reported in the inset boxes. Asterisks indicate statistically significant differences by pairwise comparison between sexes at specific timepoints: *p < 0.05, **p < 0.01 and ***p < 0.001. All three strength scores showed a significant age‐related decline, while SS2 demonstrated the highest sensitivity in detecting sex‐related differences.

The results revealed that all SS maintained a strong association with age (*p* < 0.001), with consistent trends observed in both male and female mice. Interestingly, the SS2 showed enhanced sensitivity in detecting sex differences (*p* = 0.001), outperforming both the SS5 (*p* = 0.057) and the SS3 (*p* = 0.021) (Figure 5). The dot plot of the different strength scores (SS) is shown in Figure S6. The significance of sex differences across months is presented in a Table S5.

In order to account for potential health‐related confounders affecting functional decline, we performed the analysis including tumours and distended abdomen (most often due to hepatosplenomegaly, neoplasia or ascites) detected during the assessments as confounding factors.

Analyses of individual grip strength methods, as well as the composite scores SS5, SS3 and SS2, performed using generalized linear mixed models are detailed in Figures S7 and S8.

The inclusion of these variables did not substantially alter the main outcomes observed in their absence, as shown in Figure S7 for the individual grip methods and Figure S8 for the composite strength scores. However, differential sensitivity to these pathological conditions was observed among the individual strength tests: Both the BG and CG were affected by the presence of a distended abdomen. Despite the consistently significant impact of the distended abdomen on the composite strength scores (SS5, SS3 and SS2), these scores maintained strong correlations with age and preserved their ability to discriminate between sexes. These findings confirm the robustness of the composite scores in detecting biologically relevant variation, even in the presence of potentially confounding pathological conditions.

## 4 Discussion

In this study, we aimed to enhance the robustness and precision of methodologies used to assess grip strength in geriatric preclinical research. The longitudinal design of our investigation allowed for the continuous monitoring of the same cohort of mice, providing valuable insights into how various grip strength measurement techniques reflect age‐related physical decline. We examined mice starting from an advanced age of 24 months and tracked them to an exceptionally old age of 35 months, which parallels the lifespan of a centenarian [33]. Notably, while previous research has assessed grip strength in wild‐type aged mice up to 24–26 months [28, 34, 35] and accelerated senescence models after interventions such as exercise training and cell therapies [36, 37], our study significantly extends the observational timeline in wild‐type cohorts, enabling detailed characterization of muscle function decline during late stages of aging, which have been infrequently addressed in previous studies.

We employed a comprehensive set of four criteria throughout our study to ensure a robust and valid quantitative analysis of grip strength measurements. In the first criterion, we assessed the relationship between grip strength score and age, demonstrating that all grip strength measurement methods effectively detected the decline in muscle function over time. Second, we evaluated the ability of our measurements to distinguish between male and female groups. This point is particularly critical given that most literature on strength function predominantly focuses on male populations [38], potentially overlooking important sex‐specific differences in strength dynamics. The methods that best describe sex differences were BG and MG. Third, we emphasized the importance of data repeatability across the five different methods, which ensured that our findings remained consistent and reliable across different timepoints and experimental conditions. All methods exhibited a coefficient of variation (CV%) ranging from 7% to 17%, indicating good repeatability in the grip strength measurements. Variability analysis, including intragroup and interbatch comparisons of CV%, revealed no significant differences between the methods. The observed consistency across batches further supports the repeatability and accuracy of the data, suggesting that the methods are robust and capable of providing reliable measurements over time. These results reinforce the methodologies' validity, ensuring their effectiveness for repeatable and accurate longitudinal assessments in geriatric preclinical research.

Lastly, we explored the association between grip strength score and muscle size; understanding this correlation can provide deeper insights into how muscle mass influences physical strength throughout aging. Muscle size was measured using callipers, providing a noninvasive in vivo method for assessing skeletal muscle dimensions, with MGs and GGs showing the highest correlation coefficients.

The decline in muscle strength with age is generally more pronounced than changes in muscle size. Previous studies suggest that muscle mass may not experience dramatic reductions with aging, whereas muscle function and strength are significantly compromised [39]. Muscle size decreases less markedly over time; however, the mice develop muscle wasting, likely due to age‐related disease such as tumours, which significantly contributes to the decline in strength.

The composite scores and grip strength methods consistently exhibit strong correlations with age and maintain their capacity to effectively discriminate between sexes, despite the significant influence of distended abdomen. However, it is important to acknowledge that these observed effects may be partially confounded by the presence of tumours or ascites, which frequently go undetected during routine visual examinations.

In aged mice, changes in body weight can result from factors such as tumour development, ascites, hepatosplenomegaly and severe muscle wasting associated with sarcopenia. These conditions introduce in geriatric mice a substantial variability in weight measurements unrelated to actual muscle function, compromising the reliability of the normalization of strength assessments to body weight. While normalization to body weight is generally suitable for younger or adult [24, 25, 35, 36, 37, 40, 41] where body composition is relatively stable, it presents challenges in geriatric mice, potentially distorting evaluations of proper muscle function. Consequently, refraining from normalization in aged mice may provide a more accurate representation of muscle performance, effectively mitigating the influence of weight fluctuations on strength assessments. From a preclinical research perspective, this finding is particularly relevant for the design of intervention studies aimed at improving or preserving muscle function in late life. Applying a longitudinal approach and avoiding normalization in geriatric cohorts can enable more reliable comparisons across treatment arms, especially when testing interventions such as exercise training, surgical, pharmacologic and diet interventions that may differentially affect body composition and functional strength [12]. This has implications for defining functional endpoints and therapeutic windows in preclinical trials of sarcopenia and frailty, particularly when seeking to translate findings to human studies that rely on absolute measures such as handgrip strength.

In a previous study, we demonstrated the feasibility of combining all five parameters described here into a single variable that effectively represents strength (Marcozzi et al.) [19]. However, recognizing that certain methods outperform others, we sought to investigate whether simplifying the strength score by utilizing a subset of methodologies could improve the reproducibility of measurements across laboratories by offering a more streamlined and easily replicable approach in diverse experimental settings. These newly created SS2 and SS3 maintain a strong correlation with the age of the SS5, established by Marcozzi et al. [19], while reducing the number of methods employed. Specifically, the SS3 strength scoring system retains CG, a simple and reproducible measure suitable for any laboratory. MG has demonstrated a good capability in differentiating between sexes and correlating with muscle size, and BG shows a strong correlation and excellent measurement reliability across sexes. Moreover, we also described an SS2 composed solely of weights and the MG and BG, which significantly improves the detection of sex differences, making it particularly valuable for future research endeavours.

Additionally, they enhance the ability to detect sex‐based differences, making them particularly valuable for studies focused on populations balanced for sex. This streamlined approach simplifies in vivo procedures and preserves critical evaluative strength, thereby enhancing and optimizing longitudinal preclinical studies to screen new therapeutic drugs. Furthermore, this methodology enables us to connect preclinical findings with clinical applications, ensuring that insights gained from animal models are successfully translated into human populations, thereby accelerating the development of geriatric therapies. This strength score may prove valuable in models of neurodegenerative diseases such as Parkinson's disease, Huntington's chorea, muscular dystrophy and amyotrophic lateral sclerosis (ALS), providing a quantitative and sensitive assessment of motor and muscle decline, which is crucial for monitoring disease progression and evaluating therapeutic interventions [20, 42, 43].

While grip strength is typically assessed using single‐method approaches [44], integrating multiple grip strength methodologies into composite scoring systems provides more robust and reproducible tools that enhance the sensitivity and reliability of muscle function assessment at the cost of increased workload [19]. The simplified composite scores developed in this study overcome this limitation by maintaining robustness and reproducibility while significantly reducing the testing burden. These scores have the potential to detect subtle functional improvements in a wide range of future intervention studies (e.g., physical exercise, senotherapeutics, anabolic agents or gene therapies).

The increasing global life expectancy necessitates the development of geriatric drugs to address the complex health challenges of older adults, particularly those related to age‐related conditions like sarcopenia and frailty. At the same time, sarcopenia may also result as a side effect of several drugs. As polypharmacy becomes more prevalent, with older adults managing multiple chronic conditions, there is a critical need for treatments that not only address these issues but also minimize adverse effects on skeletal muscle health [45]. Thus, a robust preclinical model that allows identifying the loss of muscle function may be helpful also in the assessment of drug safety. The timing of preclinical evaluations is critical for accurately assessing the responses of frail and sarcopenic phenotypes [46, 47]. To obtain meaningful insights, it is essential to conduct these assessments during the late and geriatric stages of aging, as demonstrated in these studies.

This study presents several limitations that should be considered when interpreting the findings. First, the analysis was conducted exclusively on aged C57BL/6 mice. While this strain is a well‐established model for aging research, it would be valuable to extend the investigation to models of accelerated senescence, as well as genetically heterogeneous populations like HET3 mice, to enhance the generalizability and translational relevance of the results.

Moreover, although grip strength assessments and scoring methods provide important functional readouts, they reflect only one aspect of musculoskeletal health. This study did not include complementary evaluations such as muscle histology, metabolic profiling or in vivo imaging, which could offer deeper mechanistic insights and a more comprehensive understanding of age‐related muscle decline.

In conclusion, the defined strength scores are robust tools for evaluating drug efficacy related to physical strength, ensuring precise and reliable results in this context. Given the complexity of health challenges in older adults, developing reliable preclinical models and SS is essential for accurately evaluating both the efficacy and safety of new pharmacological interventions. The new scores we propose are specifically designed to address the aging population's needs. These scores facilitate a more precise evaluation of a drug's effectiveness and ensure that any adverse effects on skeletal muscle are identified early in the preclinical phase. This dual focus on efficacy and safety is particularly crucial, considering the heightened susceptibility of older adults to muscle‐related side effects.

Furthermore, these scores can be valuable for assessing the impact of targeted physical exercise training therapies aimed at enhancing muscle strength, which represent a crucial nonpharmacological strategy for mitigating sarcopenia‐related physical frailty and improving functional independence in the aging population.

## Conflicts of Interest

The authors declare no conflicts of interest.

## Supporting information


**Figure S1:** Schematic representation of the five methods used to evaluate grip strength in aged mice. Deacon (DG), Modified Deacon (MG), Cage Lift Measurements (CG), Grid Strength Meter (GG) and Bar Strength Meter (BG).
**Figure S2:** Survival curves of the study population. (A) Kaplan–Meier survival curve with Log‐Rank test of male (blue line, *n* = 70) and female (red line, *n* = 77) C57BL/6 mice. Age is expressed in months. **(B)** Cox regression survival curve of male (blue line, *n* = 70) and female (red line, *n* = 70) C57BL/6 mice, adjusted for confounding variables (sex and experimental batch). Both survival curves represent only mice that died naturally, excluding 13 mice used for organ explants.
**Figure S3:** Raw grip strength measurements and their association with age, including regression lines and *R* ^2^ coefficient. The figure presents scatter plots illustrating the relationship between age and grip strength measurements obtained using five different methods in a longitudinal cohort of C57BL/6J mice (*n* = 160), with a total of 782 measurements collected over time. Each plot displays the full dataset, including all timepoints, with fitted linear regression lines and corresponding *R* ^2^ values reported to indicate the strength of the association. Grip strength was measured using the following methods: Deacon (DG), Modified Deacon (MG), Cage Lift (CG), Grid Strength Meter (GG) and Bar Strength Meter (BG).
**Figure S4:** Correlation of age with grip strength normalized by body weight. Correlation analysis was conducted in a longitudinal cohort of C57BL/6J mice (*n* = 160), for a total of 782 grip strength measurements collected over time. Panels A–E show scatter plots illustrating the relationship between age and normalized grip strength scores obtained using the following methods: Deacon Grip normalized to body weight (DGbw, A), Modified Grip (MGbw, B), Cage Lift (CGbw, C), Grid Strength meter (GGbw, D) and Bar Strength meter (BGbw, E). Each panel displays the full dataset, the fitted linear regression line and the corresponding *R* ^2^ value. Statistical analysis was performed using two approaches: (1) Spearman's rank correlation coefficient (r_s_) with 95% confidence intervals (CIs) and *p*‐values; (2) Linear mixed‐effects models (LMMs), which account for repeated measures across time, providing slope estimates with corresponding 95% CIs and *p*‐values. All methods showed a significant inverse correlation between normalized grip strength and age.
**Figure S5:** Representative aged mouse exhibiting pronounced weight loss. The trends of Absolute Grip Strength Score and GripBW scores follow significantly different patterns. In particular, the GripBW scores show markedly different trends.
**Figure S6:** Association of Age with SS5, SS3 and SS2 The figure shows scatter plots illustrating the relationship between age and three composite grip strength scores: SS5, SS3 and SS2, in a longitudinal cohort of C57BL/6J mice (*n* = 160), with a total of 782 grip strength measurements collected over time. Each plot displays the full dataset, including all timepoints, with fitted linear regression lines and corresponding *R* ^2^ values reported to indicate the strength of the association.
**Figure S7:** Analysis of grip strength methods adjusted for confounding pathological conditions Quantitative analysis of five grip strength measurements in a cohort of C57BL/6J mice (*n* = 160), longitudinally monitored for a total of 782 repeated measurements. Panels A–E show the age‐related trajectories of: (A) Deacon Grip Strength (DG), (B) Modified Grip Strength (MG), (C) Cage Lift (CG), (D) Grid Strength meter (GG) and (E) Bar Strength meter (BG), plotted as a function of age (in months) for male (blue) and female (red) mice. Values are presented as model‐derived mean estimates with 95% confidence intervals, obtained using a Generalized Linear Mixed Model (GLMM) for longitudinal data. The model included sex, age, experimental batch and pathological conditions (presence of tumours and/or distended abdomen) as fixed effects. *P*‐values for the fixed effects of sex and age are reported in the insets. Asterisks indicate statistically significant differences between sexes at specific timepoints (*p < 0.05, **p < 0.01, ***p < 0.001 and ****p < 0.0001).
**Figure S8:** Analysis of Composite Strength Scores (SS5, SS3 and SS2) adjusted for confounding pathological conditions. C57BL/6J mice (*n* = 160) were longitudinally monitored for grip strength, resulting in a total of 782 repeated observations. Composite strength scores were calculated using different combinations of strength assessment methods:(A) SS5, including Deacon Grip Strength (DG), Modified Grip Strength (MG), Cage Lift (CG), Grid Strength meter (GG) and Bar Strength meter (BG); (B) SS3, composed of MG, CG and BG; (C) SS2, based on MG and BG only. Scores are plotted as a function of age for male (blue) and female (red) mice. Data represent model‐derived mean estimates with 95% confidence intervals, obtained using a Generalized Linear Mixed Model (GLMM) for longitudinal data. Sex, age (in months), batch and pathological conditions (the presence of tumours and/or distended abdomen) were included as fixed effects. *P*‐values for sex and age are reported in the inset boxes. Asterisks indicate statistically significant sex differences at specific timepoints (*p < 0.05, **p < 0.01 and ***p < 0.001). All three composite scores exhibited significant age‐related decline, and SS2 showed the highest sensitivity for detecting sex‐related differences.
**Table S1:** Mouse population characteristics.
**Table S2:** Distribution by age of the total number of mice with measured grip strength.
**Table S3:** Significance of sex differences across months for each grip strength measurement. Grip strength methods: Deacon (DG), Modified Deacon (MG), Cage Lift (CG), Grid Strength Meter (GG) and Bar Strength Meter (BG).
**Table S4:** Interbatch variability for each grip strength method. The table reports the mean, standard deviation (SD) and coefficient of variation (CV%) for each grip strength method across two experimental batches, along with the number of mice (*n*) assessed at 24, 25 and 26 months of age in a longitudinal cohort of 160 C57BL/6J mice. Specifically, in Batch 1, the number of mice assessed was 59 at 24 months, 103 at 25 months and 79 at 26 months; in Batch 2, the corresponding numbers were 31, 47 and 63, respectively. Interbatch variability was evaluated using the coefficient of variation (CV%) as the primary statistical parameter. Grip strength was measured using the following methods: Deacon Grip Strength (DG), Modified Grip Strength (MG), Cage Lift (CG), Grid Strength (GG) and Bar Strength (BG).
**Table S5:** Significance of sex differences across months for each strength score.

## Data Availability

The data that support the findings of this study are available from the corresponding author (Marco Malavolta) upon reasonable request.
