# Correction to “A Comparative Analysis of Grip Strength Evaluation Methods in a Large Cohort of Aged Mice”

**DOI:** 10.1002/jcsm.70077

**Published:** 2025-09-23

Bigossi G , Marcozzi S , Giuliani ME , Lai G , Bartozzi B , Orlando F , Gerosa L , Malvandi AM , Putavet D , Bouma E , de Keizer PL , Lombardi G , Malavolta M . “A Comparative Analysis of Grip Strength Evaluation Methods in a Large Cohort of Aged Mice,” Journal of Cachexia, Sarcopenia and Muscle 16, no. 5 (2025): e70050, 10.1002/jcsm.70050.40873209 PMC12391727

One affiliation for Giovanni Lombardi is missing. The complete affiliation list for the author is as follows:

- Laboratory of Experimental Biochemistry and Advanced Diagnostics, IRCCS Ospedale Galeazzi‐Sant'Ambrogio, Milan, Italy
- Department of Athletics, Strength and Conditioning, Poznań University of Physical Education, Poznań, Poland

We apologize for this error.

