# Supplementary material for: A Comparative Analysis of Grip Strength Evaluation Methods in a Large Cohort of Aged Mice
Source: J Cachexia Sarcopenia Muscle. 2025 Aug 27;16(5):e70050. doi: 10.1002/jcsm.70050 (PMC12391727; doi:10.1002/jcsm.70050)
Supplement: Supplementary file 1 — Figure S1: Schematic representation of the five methods used to evaluate grip strength in aged mice. Deacon (DG), Modified Deacon (MG), Cage Lift Measurements (CG), Grid Strength Meter (GG) and Bar Strength Meter (BG). Figure S2: Survival curves of the study population. (A) Kaplan–Meier survival curve with Log‐Rank test of male (blue line, n = 70) and female (red line, n = 77) C57BL/6 mice. Age is expressed in months. (B) Cox regression survival curve of male (blue line, n = 70) and female (red line, n = 70) C57BL/6 mice, adjusted for confounding variables (sex and experimental batch). Both survival curves represent only mice that died naturally, excluding 13 mice used for organ explants. Figure S3: Raw grip strength measurements and their association with age, including regression lines and R 2 coefficient. The figure presents scatter plots illustrating the relationship between age and grip strength measurements obtained using five different methods in a longitudinal cohort of C57BL/6J mice (n = 160), with a total of 782 measurements collected over time. Each plot displays the full dataset, including all timepoints, with fitted linear regression lines and corresponding R 2 values reported to indicate the strength of the association. Grip strength was measured using the following methods: Deacon (DG), Modified Deacon (MG), Cage Lift (CG), Grid Strength Meter (GG) and Bar Strength Meter (BG). Figure S4: Correlation of age with grip strength normalized by body weight. Correlation analysis was conducted in a longitudinal cohort of C57BL/6J mice (n = 160), for a total of 782 grip strength measurements collected over time. Panels A–E show scatter plots illustrating the relationship between age and normalized grip strength scores obtained using the following methods: Deacon Grip normalized to body weight (DGbw, A), Modified Grip (MGbw, B), Cage Lift (CGbw, C), Grid Strength meter (GGbw, D) and Bar Strength meter (BGbw, E). Each panel displays the full dataset, the fitted [file JCSM-16-e70050-s001.docx]

**A Comparative Analysis of Grip Strength Evaluation Methods in a Large Cohort of Aged Mice**

Giorgia Bigossi ^1^, Serena Marcozzi ^1^, Maria Elisa Giuliani ^1^, Giovanni Lai ^1^, Beatrice Bartozzi ^2^, Fiorenza Orlando ^3^, Laura Gerosa ^4^, Amir Mohammad Malvandi ^4^, Diana Putavet ^5^, Esmée Bouma ^5^, Peter L. J. de Keizer ^5,6^, Giovanni Lombardi ^4^, Marco Malavolta ^1,7*^

**Affiliations**

1 Advanced Technology Center for Aging Research and Geriatric Mouse Clinic, IRCCS INRCA, 60121 8 Ancona, Italy

2 Advanced Technology Center for Aging Research, IRCCS INRCA, 60121 Ancona, Italy

3 Experimental Animal Models for Aging Unit, Scientific Technological Area, IRCCS INRCA, 60015 12 Falconara Marittima (AN), Italy

4 Laboratory of Experimental Biochemistry & Advanced Diagnostics, IRCCS Ospedale Galeazzi-Sant'Ambrogio, Via Cristina Belgioioso 173, 20157, Milan, Italy.

5 Cleara Biotech B.V., Utrecht, The Netherlands

6 Center for Molecular Medicine, Division of Laboratories, Pharmacy and Biomedical Genetics, University Medical Center Utrecht, Universiteitsweg 100, 3584CG, Utrecht, The Netherlands

7 Department of Clinical and Molecular Sciences (DISCLIMO), Università Politecnica delle Marche, 60126 Ancona, Italy

* Correspondence to: Marco Malavolta, Advanced Technology Center for Aging Research, IRCCS 25 INRCA, 60121 Ancona, Italy. **Email:** [m.malavolta@inrca.it](mailto:m.malavolta@inrca.it)


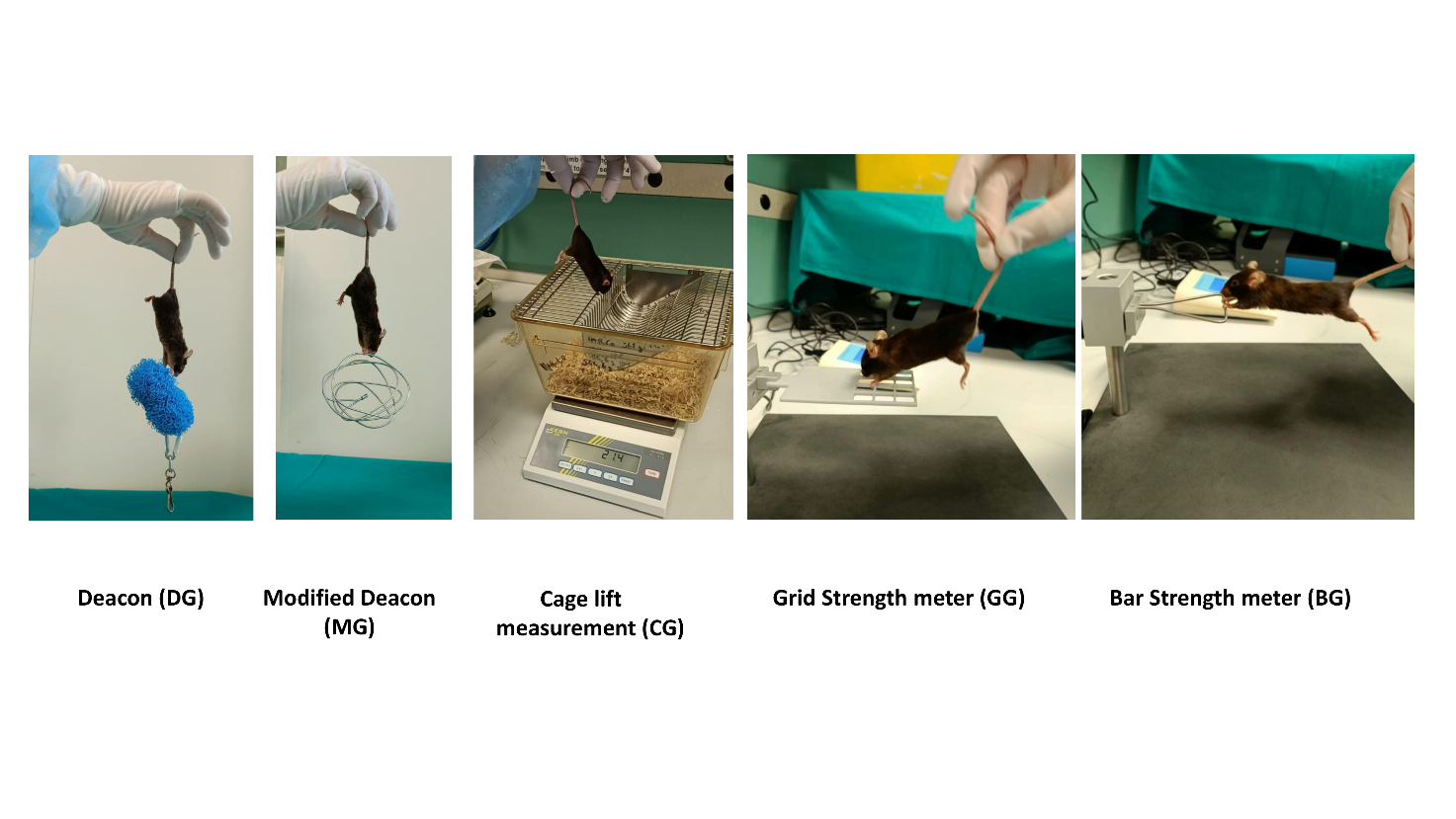
**Figure S1. Schematic representation of the five methods used to evaluate grip strength in aged mice.**

**Deacon (DG), Modified Deacon (MG), Cage Lift Measurements (CG), Grid Strength Meter (GG), and Bar Strength Meter (BG).**

**A**

**B**

**p=0.918**

**p=0.600**

**Figure S2. Survival Curves of the study population. (A)** Kaplan-Meier survival curve with Log-Rank test of male (blue line, n=70) and female (red line, n=77) C57BL/6 mice. Age is expressed in months. **(B)** Cox regression survival curve of male (blue line, n=70) and female (red line, n=70) C57BL/6 mice, adjusted for confounding variables (sex and experimental batch). Both survival curves represent only mice that died naturally, excluding thirteen mice used for organ explants.

**R^2^ linear =0.139**

**R^2^ linear =0.248**

**A**

**B**

**R^2^ linear =0.119**

**C**

**D**

**E**

**R^2^ linear =0.275**

**R^2^ linear =0.251**

**Figure S3. Raw Grip Strength Measurements and Their Association with Age, Including Regression Lines and R² coefficient. The figure presents scatter plots illustrating the relationship between age and grip strength measurements obtained using five different methods in a longitudinal cohort of C57BL/6J mice (n = 160), with a total of 782 measurements collected over time. Each plot displays the full dataset, including all timepoints, with fitted linear regression lines and corresponding R² values reported to indicate the strength of the association.** **Grip strength was measured using the following methods: Deacon (DG), Modified Deacon (MG), Cage Lift (CG), Grid Strength Meter (GG), and Bar Strength Meter (BG).**


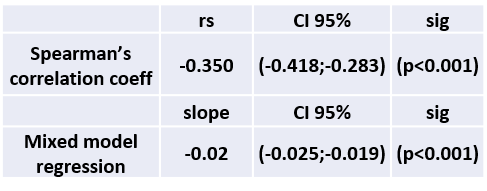

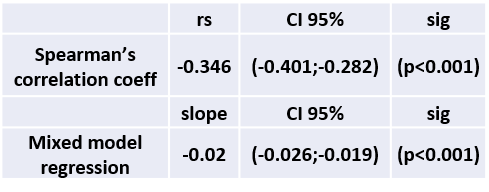

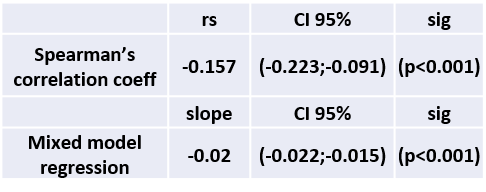

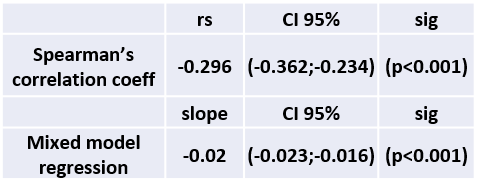


**R^2^ linear =0.042**

**R^2^ linear =0.106**

**A**

**B**

**C**

**D**

**R^2^ linear =0.135**

**R^2^ linear =0.133**

**R^2^ linear =0.143**

**E**


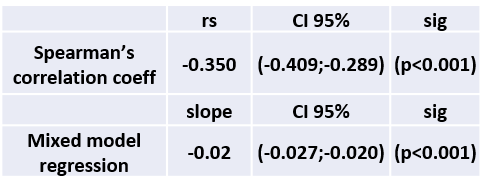


**Figure S4. Correlation of Age with Grip Strength Normalized by Body Weight.**

**Correlation analysis was conducted in a longitudinal cohort of C57BL/6J mice (n = 160), for a total of 782 grip strength measurements collected over time. Panels A–E show scatter plots illustrating the relationship between age and normalized grip strength scores obtained using the following methods: Deacon Grip normalized to body weight (DGbw, A), Modified Grip (MGbw, B), Cage Lift (CGbw, C), Grid Strength meter (GGbw, D), and Bar Strength meter (BGbw, E). Each panel displays the full dataset, the fitted linear regression line, and the corresponding R² value. Statistical analysis was performed using two approaches: (1) Spearman’s rank correlation coefficient (rₛ) with 95% confidence intervals (CIs) and p values;(2) Linear mixed-effects models (LMMs), which account for repeated measures across time, providing slope estimates with corresponding 95% CIs and p values. All methods showed a significant inverse correlation between normalized grip strength and age.**


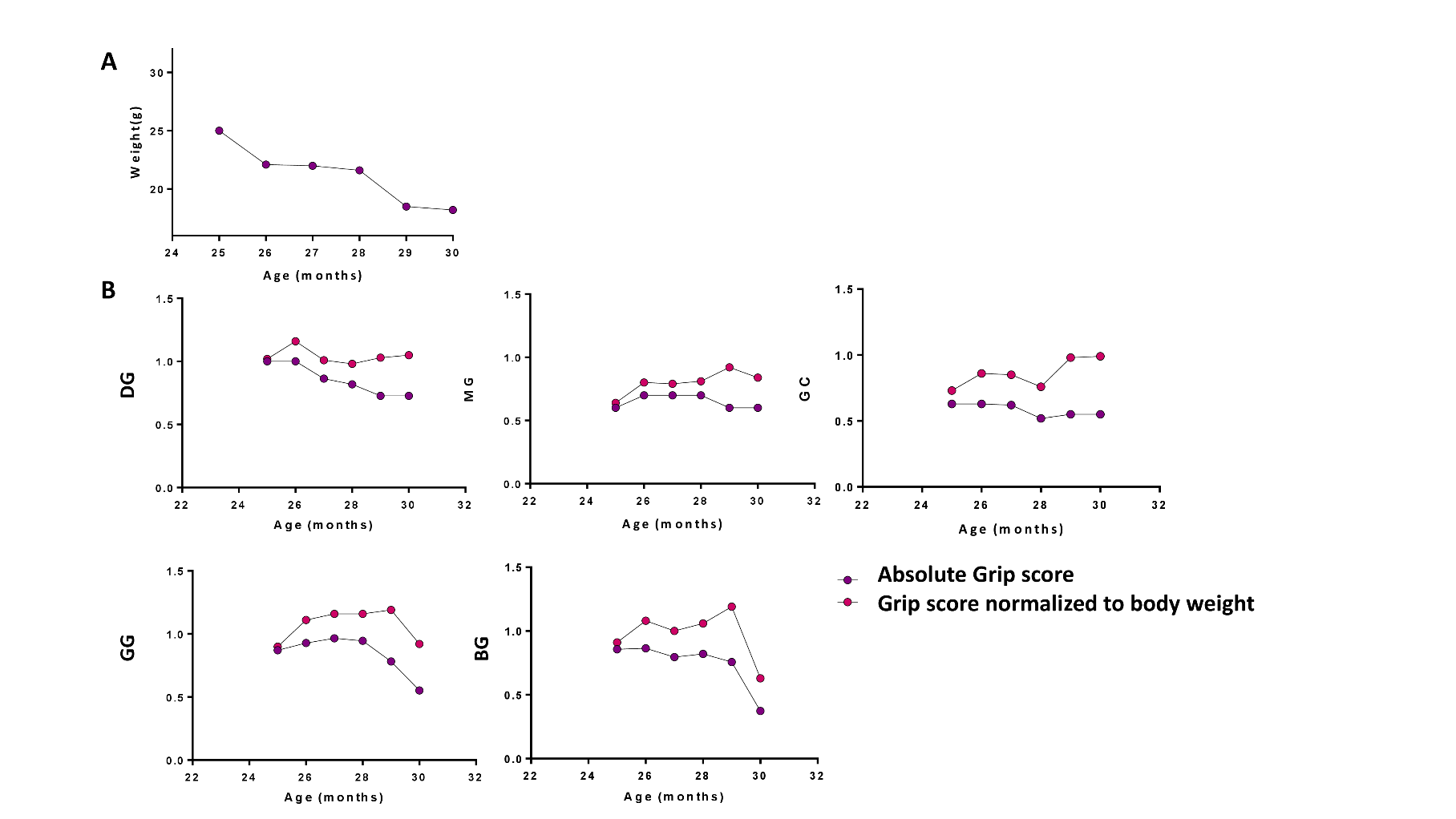


**Figure S5. Representative aged mouse exhibiting pronounced weight loss.** The trends of Absolute Grip Strength Score and GripBW scores follow significantly different patterns. In particular, the GripBW scores show markedly different trends.


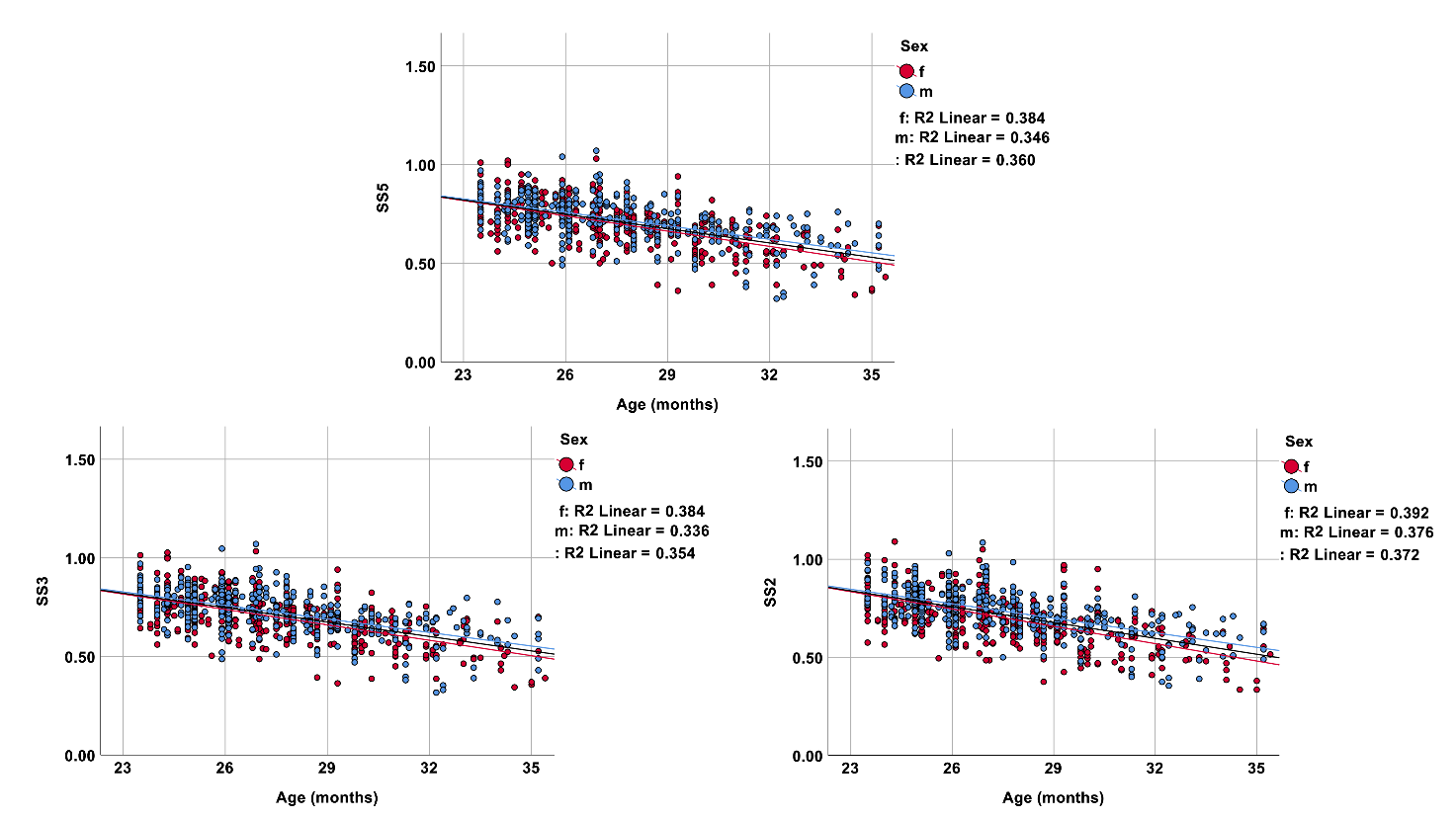


**Figure S6. Association of Age with SS5, SS3, and SS2 The figure shows scatter plots illustrating the relationship between age and three composite grip strength scores: SS5, SS3, and SS2, in a longitudinal cohort of C57BL/6J mice (n = 160), with a total of 782 grip strength measurements collected over time. Each plot displays the full dataset, including all timepoints, with fitted linear regression lines and corresponding R² values reported to indicate the strength of the association.**


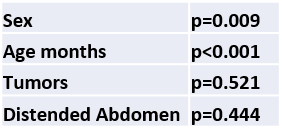

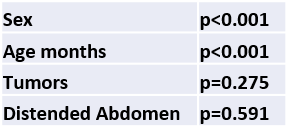

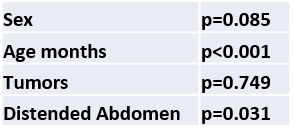

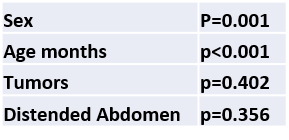


**A**

**B**

**C**

**D**

**E**

***

*

*

*

*

*

*

*

**

*

*

*

*

*

*

*

*

*

***

***

***

**

**

*


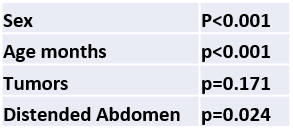


**Figure S7. Analysis of grip strength methods adjusted for confounding pathological conditions**

**Quantitative analysis of five grip strength measurements in a cohort of C57BL/6J mice (n = 160), longitudinally monitored for a total of 782 repeated measurements. Panels A–E show the age-related trajectories of: (A) Deacon Grip Strength (DG), (B) Modified Grip Strength (MG), (C) Cage Lift (CG), (D) Grid Strength meter (GG), and (E) Bar Strength meter (BG), plotted as a function of age (in months) for male (blue) and female (red) mice. Values are presented as model-derived mean estimates with 95% confidence intervals, obtained using a Generalized Linear Mixed Model (GLMM) for longitudinal data. The model included sex, age, experimental batch, and pathological conditions (presence of tumors and/or distended abdomen) as fixed effects. P-values for the fixed effects of sex and age are reported in the insets. Asterisks indicate statistically significant differences between sexes at specific time points (*p < 0.05, **p < 0.01, ***p < 0.001, ****p < 0.0001).**


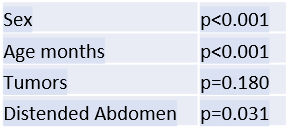

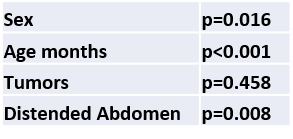

**A**

*

*

**

**B**

*

*

*


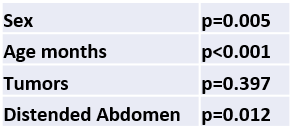

*

*

*

***

*

**

**C**


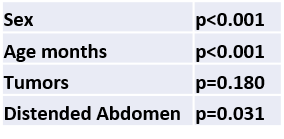


**Figure S8. Analysis of Composite Strength Scores (SS5, SS3, SS2) adjusted for confounding pathological conditions. C57BL/6J mice (n = 160) were longitudinally monitored for grip strength, resulting in a total of 782 repeated observations. Composite strength scores were calculated using different combinations of strength assessment methods:(A) SS5, including Deacon Grip Strength (DG), Modified Grip Strength (MG), Cage Lift (CG), Grid Strength meter (GG), and Bar Strength meter (BG); (B) SS3, composed of MG, CG, and BG; (C) SS2, based on MG and BG only. Scores are plotted as a function of age for male (blue) and female (red) mice. Data represent model-derived mean estimates with 95% confidence intervals, obtained using a Generalized Linear Mixed Model (GLMM) for longitudinal data. Sex, age (in months), batch, and pathological conditions (presence of tumors and/or distended abdomen) were included as fixed effects. P-values for sex and age are reported in the inset boxes. Asterisks indicate statistically significant sex differences at specific time points (*p < 0.05, **p < 0.01, ***p < 0.001). All three composite scores exhibited significant age-related decline, and SS2 showed the highest sensitivity for detecting sex-related differences.**

**Table S1. Mouse Population Characteristics**

|  | **All** | **Males** | **Females** |
| --- | --- | --- | --- |
| N | 160 | 76 | 84 |
| N recorded deaths (n censored) | 147 (13) | 70 (6) | 77 (7) |
| age at enrolment (Mean ± SD) | 24.64 ± 0.48 | 24.67 ± 0.47 | 24.62 ± 0.48 |
| age at death  (Mean ± SD) | 29.16 ± 3.54 | 28.99 ± 3.33 | 29.31 ± 3.77 |
| N follow-up  (Mean ± SD) | 6.84± 2.99 | 6.80 ± 3.00 | 6.87 ± 2.98 |

**Table S2 Distribution by age of the total number of mice with measured grip strength**.

|  | ***Number of mice with measured grip strength*** | | |  |  |
| --- | --- | --- | --- | --- | --- |
| ***AGE (months)*** | **Total (n)** | **Males (n)** | **Females**  **(n)** | |  |
| ***24*** | 90 | 53 | 37 | |  |
| ***25*** | 150 | 70 | 80 | |  |
| ***26*** | 126 | 55 | 71 | |  |
| ***27*** | 100 | 56 | 44 | |  |
| ***28*** | 88 | 41 | 47 | |  |
| ***29*** | 66 | 30 | 36 | |  |
| ***30*** | 54 | 26 | 28 | |  |
| ***31*** | 37 | 16 | 21 | |  |
| ***32*** | 29 | 15 | 14 | |  |
| ***33*** | 19 | 10 | 9 | |  |
| ***34*** | 13 | 6 | 7 | |  |
| ***35*** | 10 | 6 | 4 | |  |
| ***36*** | 6 | 3 | 3 | |  |
| ***37*** | 2 | 2 | 0 | |  |
| ***38*** | 1 | 1 | 0 | |  |

**
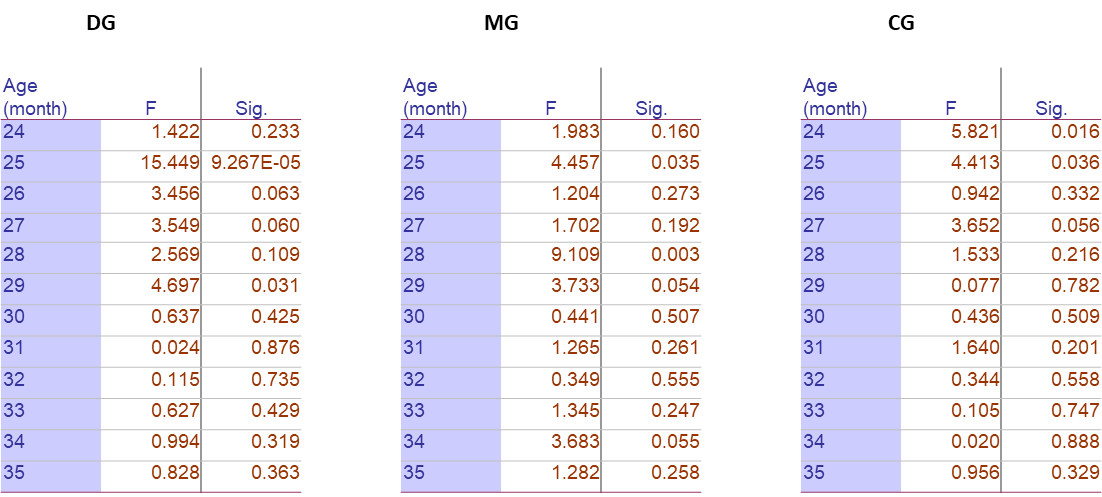
Table S3. Significance of sex differences across months for each grip strength measurement**. **Grip strength methods: Deacon (DG), Modified Deacon (MG), Cage Lift (CG), Grid Strength Meter (GG), and Bar Strength Meter (BG).**

**
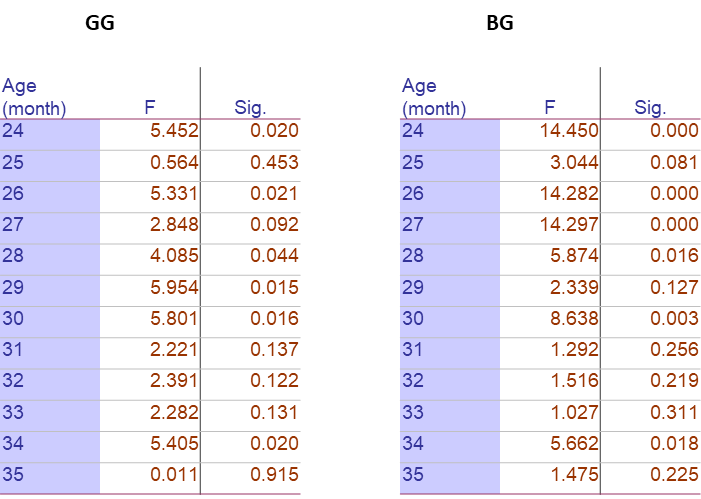
**

**Table S4. Inter-batch variability for each grip strength method.**

**The table reports the mean, standard deviation (SD), and coefficient of variation (CV%) for each grip strength method across two experimental batches, along with the number of mice (n) assessed at 24, 25, and 26 months of age in a longitudinal cohort of 160 C57BL/6J mice. Specifically, in batch 1, the number of mice assessed was 59 at 24 months, 103 at 25 months, and 79 at 26 months; in batch 2, the corresponding numbers were 31, 47, and 63, respectively. Inter-batch variability was evaluated using the coefficient of variation (CV%) as the primary statistical parameter. Grip strength was measured using the following methods: Deacon Grip Strength (DG), Modified Grip Strength (MG), Cage Lift (CG), Grid Strength (GG), and Bar Strength (BG).**

| **BATCH1** | **DG** | **MG** | **CG** | **GG** | **BG** | **(n)** |
| --- | --- | --- | --- | --- | --- | --- |
| **24** | 15.31 | 16.57 | 10.16 | 7.50 | 11.37 | 59 |
| **25** | 16.75 | 14.80 | 14.09 | 10.88 | 12.13 | 103 |
| **26** | 14.91 | 15.36 | 11.66 | 13.13 | 14.74 | 79 |
| **BATCH 2** | **DG** | **MG** | **CG** | **GG** | **BG** | **(n)** |
| **24** | 12.98 | 12.96 | 12.96 | 7.44 | 8.29 | 31 |
| **25** | 10.33 | 9.34 | 10.70 | 10.30 | 9.82 | 47 |
| **26** | 10.32 | 17.85 | 13.08 | 7.15 | 13.72 | 63 |

**Table S5. Significance of sex differences across months for each strength score.**

**
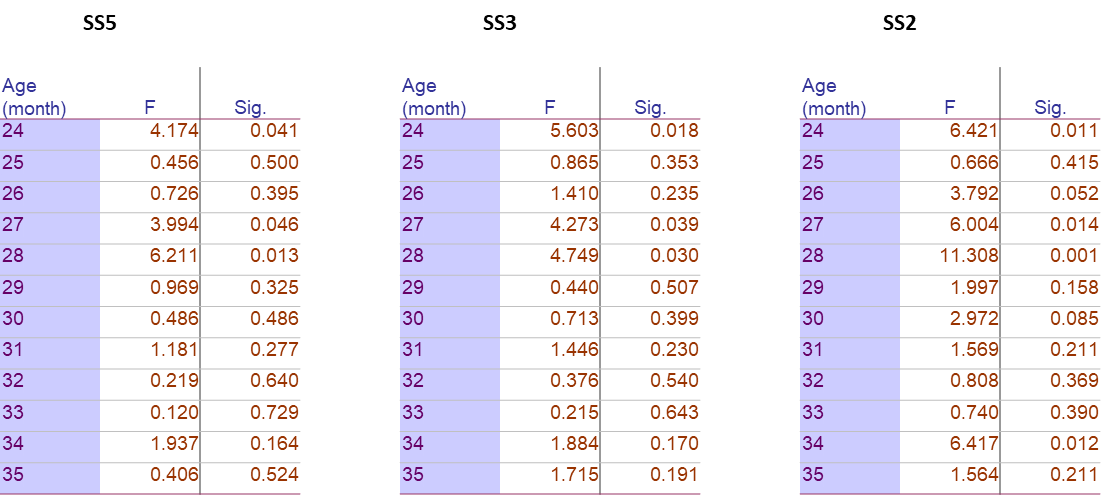
**
